# Supplementary material for: Neurological phenotypes and treatment outcomes in Eagle syndrome: systematic review and meta-analysis
Source: PeerJ. 2024 Jun 26;12:e17423. doi: 10.7717/peerj.17423 (PMC11214433; doi:10.7717/peerj.17423)
Supplement: Supplemental Information 2 [file peerj-12-17423-s002.docx]

Rationale: Eagle syndrome remains underappreciated in the neurological community. We aimed to determine the most common neurological and non-neurological clinical presentations in patients with Eagle syndrome and to assess the clinical outcome post-surgical resection in comparison to non-surgical therapies.

Contribution: Since a comprehensive and up-to-date review on Eagle syndrome has not been done previously, the paper unveils novel perspectives and data points previously unavailable in the literature.
